# Sclerotic Lesions of the Jaw: A Pictorial Review

**DOI:** 10.5334/jbsr.2208

**Published:** 2021-04-08

**Authors:** Stefaan Van Hoe, Olivier Bladt, Kris Van Der Steen, Herman Van den Eynde

**Affiliations:** 1 KU Leuven, BE; 2 OLV Hospital Aalst, BE

**Keywords:** jaw, mandible, maxilla, sclerotic lesion, radiopaque lesion

## Abstract

Sclerotic lesions of the jaw are uncommon but may be clinically relevant. In this pictorial review, the most common sclerotic lesions are discussed. Three categories of lesions are distinguished; odontogenic sclerotic lesions, non-odontogenic sclerotic lesions, and mixed lytic-sclerotic lesions. In each group, non-neoplastic conditions are discussed first, followed by benign and malignant neoplasms. For each disease a brief overview is given, including histological features, epidemiology, symptoms, typical location, imaging features, and treatment. This review emphasizes which basic observations are essential to the evaluation of sclerotic jaw lesions and what elements have to be taken into account to create a proper differential diagnosis.

## Introduction

Sclerotic jaw lesions are not rare and are frequently encountered on radiographs and computed tomography (CT). However, these lesions are often underreported, mainly because the subject is not well known to general radiologists who struggle with the imaging approach and disease entities.

In this paper, an overview is given of the most important types of lesions. Emphasis is on the distinction between odontogenic and non-odontogenic lesions. When a sclerotic lesion is observed in the jaw, attention should first be focused on whether and how the lesion is related to a tooth [1,2,3,4,5]. If a lesion is intimately associated with a tooth, the lesion is most likely odontogenic, and the number of possible diagnoses is limited. Moreover, the specific location of the lesion (e.g., periapical or pericoronal) may suggest a specific diagnosis. Lesions that are clearly not tooth related, usually indicate a lesion of osseous origin.

The following conditions are discussed in this review: (1) odontogenic sclerotic lesions (cemento-osseous dysplasia, condensing osteitis, odontoma, cementoblastoma, cemento-ossifying fibroma), (2) non-odontogenic sclerotic lesions (idiopathic osteosclerosis, torus mandibularis, fibrous dysplasia, osteoma/osteoid osteoma, metastases), and (3) mixed lytic-sclerotic lesions (osteoradionecrosis, biphosphonate-related osteonecrosis, osteomyelitis).

## Odontogenic sclerotic lesions

### Cemento-osseous dysplasia

Cemento-osseous dysplasia (COD) is the most common fibro-osseous lesion found in the jaw. It represents a group of benign lesions of unknown etiology characterized by the substitution of normal bone by fibrous tissue with newly formed mineralized structures [6,7]. Three subtypes have been described: (1) focal, (2) periapical, and (3) florid. Except for the focal variant, which is more common in white women, most lesions occur in black women and women of Asian descent, usually in the fourth or fifth decade of life.

The lesions typically do not cause any symptoms. In the early stages of the disease, a vascular fibrous stroma with osteoid and some basophilic cementoid structures can be observed. In the later stages, the stroma becomes more fibrotic. A more distinct osteoid trabeculae formation is observed with the presence of thicker curvilinear bony trabeculae, and a possible occurrence of prominent cementoid masses.

**Figure 1:**
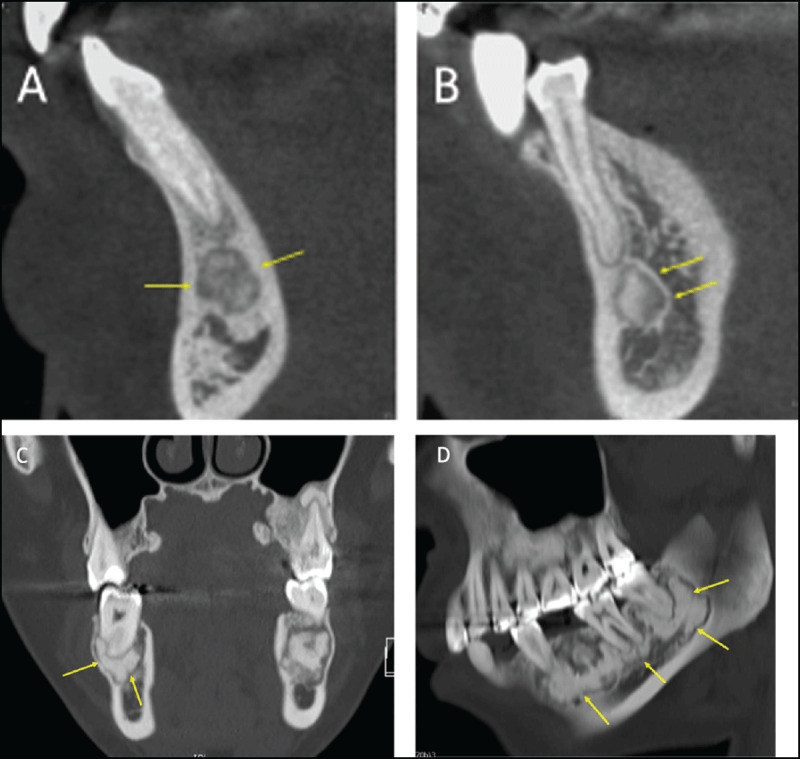
**(A)** Periapical cemento-osseous dysplasia in two different patients. CT reformations showing periapical COD lesions clearly separated from the root of the anterior tooth. The lesion in (a) has a sclerotic center and a more radiolucent peripheral part (arrows). The lesion in **(B)** has a “target appearance” with a central sclerotic part, an intermediate more lucent part, and a thin peripheral sclerotic rim (arrows). **(C, D)** Florid cemento-osseous dysplasia. On CT reformations, multiple sclerotic periapical lesions involve the mandible bilaterally; the lesions have an elliptical shape and are clearly separated from the root of the mandibular teeth (arrows).

Radiographically, all three subtypes appear initially lytic and show progression to a heterogenous sclerotic lesion, often with a radiolucent rim. The radiolucent rim may in turn be surrounded by a thin rim of sclerosis, giving the lesion a quite specific appearance (***Figure 1***). Two additional characteristics that should be noted are: (1) the lesions are typically associated with a vital-nonrestored tooth, and (2) unlike cementoblastomas, cemento-osseous dysplasia lesions do not fuse directly to the tooth root (***Figure 1***). Focal COD is typically located in the posterior quadrant of the jaw. Periapical COD is seen adjacent to a tooth-bearing area associated with one or more vital mandibular anterior teeth (***Figure 1a, b***). In florid COD, multiple lesions are observed that involve the mandible and/or maxilla bilaterally (***Figure 1c, d***). The differential diagnosis includes fibrous dysplasia, Gardner’s syndrome, and chronic osteomyelitis (see below). Asymptomatic COD needs no surgical treatment.

### Condensing osteitis

**Figure 2:**
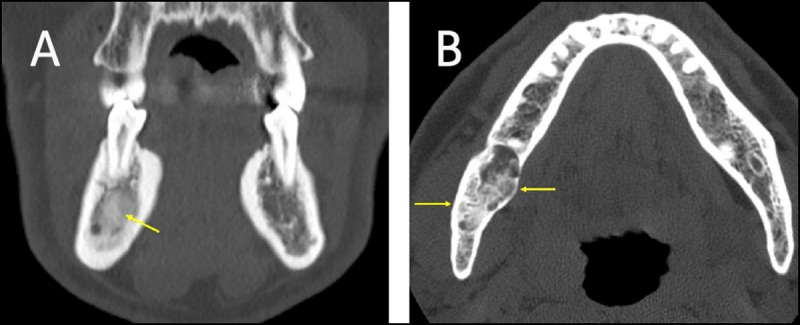
**(A)** Condensing osteitis in two patients. (a) Coronal reformation CT in a patient with history of right-sided periapical inflammation and abscess formation. A poorly marginated nonexpansile sclerotic lesion is seen (arrows). **(B)** Axial CT image obtained in a patient with longstanding caries who underwent tooth extraction. A poorly defined sclerotic lesion is seen adjacent to the extraction site (arrows).

Condensing osteitis (also known as focal sclerosing osteomyelitis) represents an asymptomatic change in osseous structure presumed to be the response to a long-standing and low-grade inflammatory stimulus from an inflamed or necrotic pulp. Histologically, dense layers of compact bone are replacing fibrofatty bone marrow and cancellous bone. On imaging, condensing osteitis is seen as a periapical, poorly marginated, nonexpansile, sclerotic lesion in the posterior mandible at the apices of the premolar or molar teeth (***Figure 2***), often associated with a carious tooth or with antecedents of root canal therapy, periodontal disease, or tooth extraction [5].

### Odontoma

**Figure 3:**
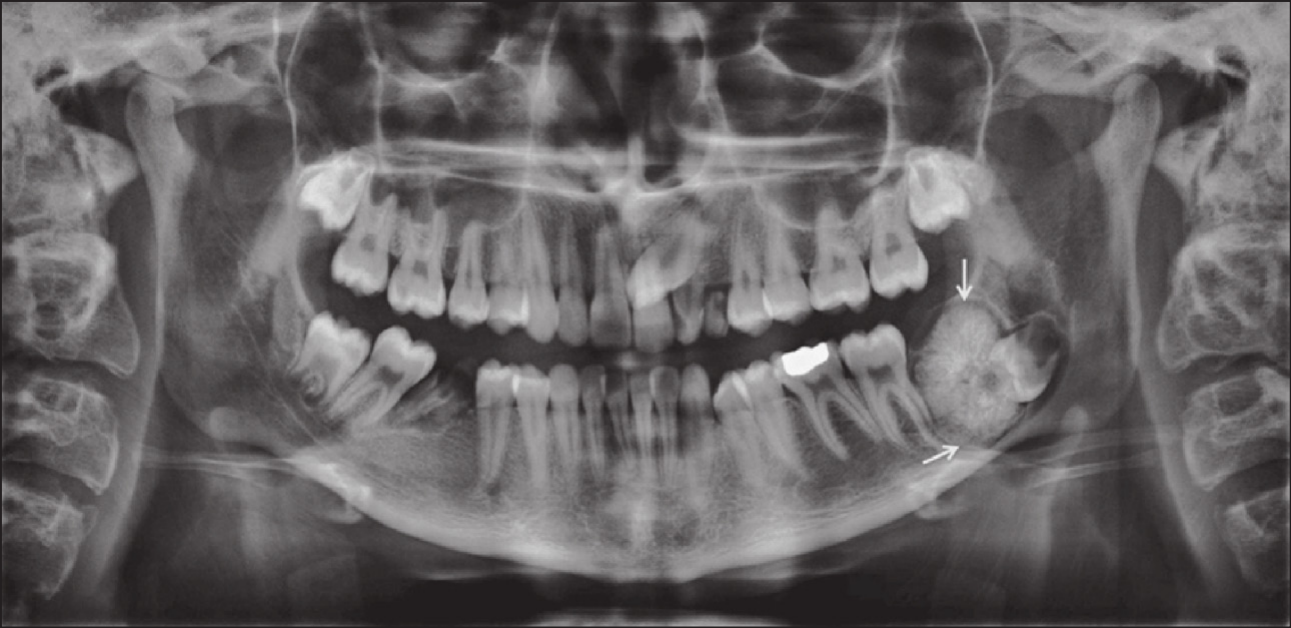
A panoramic radiograph shows a large complex odontoma associated with the impacted left mandibular third molar. Reprinted with permission from Mortazavi, Hamed & Baharvand, Maryam. (2016). Jaw lesions associated with impacted tooth: A radiographic diagnostic guide. Imaging Science in Dentistry. 46. 147. 10.5624/isd.2016.46.3.147.

Odontomas are the most common odontogenic “tumors” and actually represent a developmental-anomaly (hamartoma) [5,8]. They are most commonly seen in children and adolescents and may obstruct tooth eruption. Approximately one half of all odontomas are associated with an impacted tooth. According to the WHO 2017 Classification of Odontogenic Tumors, odontomas belong to the category of benign odontogenetic tumors, mixed epithelial and mesenchymal subtype [8,9]. They are composed of the tissues native to teeth: enamel, dentin, cementum, and pulp tissue. Odontomas are characterized by slow growth and non-aggressive behavior. Usually they are seen as an incidental radiographic finding. Subtypes of odontomas are compound odontomas and complex odontomas. Compound odontomas consist of multiple small toothlike structures called denticles and most commonly arise in the anterior maxilla. Complex odontomas appear as an amorphous hyperattenuating conglomerate mass of enamel and dentin most commonly in the molar regions the jaws. They are typically pericoronal, sharply marginated, and sclerotic, with a low-attenuation halo (***Figure 3***). The pericoronal rather than periapical position allows differentiation of odontomas from cementoblastomas. Treatment of complex odontomas consists of surgical excision.

### Cementoblastoma

**Figure 4:**
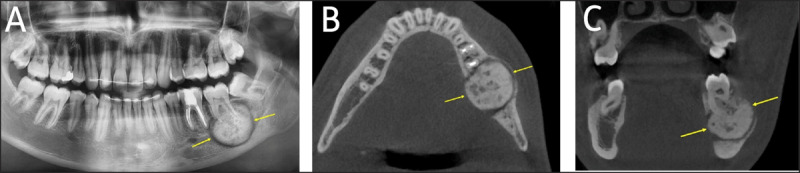
**(A)** Cementoblastoma. Panoramic radiograph of a periapical, sclerotic, sharply marginated lesion with a low-attenuation halo (arrows). The lesion is fused to the root of the tooth. **(B, C)** Cementoblastoma. Axial and coronal CT images showing a periapical, sclerotic, sharply marginated lesion with a low-attenuation halo. The lesion is fused to the root of the tooth and causes root resorption, loss of the root outline, cortical erosion, and obliteration of the periodontal ligament space (arrows).

Cementoblastoma is a rare benign odontogenic tumor of mesenchymal-ectomesenchymal origin with a typical periapical location [10]. This tumor typically occurs in patients under 25 years of age and may cause pain and or swelling of the alveolar ridges. According to the WHO 2017 Classification of Odontogenic Tumors, they belong to the category of benign odontogenetic tumors, mesenchymal subtype [8,9]. Histologically, cementoblastoma is characterized by masses of hypocellular cementum embedded in a fibrovascular stroma. At the periphery of the lesion, there is a rim of connective tissue and commonly radiating columns of cellular unmineralized tissue that accounts for the radiographic-radiolucent zone. 90% of cementoblastomas develop in the molar or premolar mandibular region. On imaging, cementoblastomas appear as a periapical, sclerotic, sharply marginated lesion with a low-attenuation halo. They directly fuse to the root of the tooth (***Figure 4***), which is an important differential diagnostic feature not seen in other periapical lesions such as cemento-osseous dysplasia and condensing osteitis. Additional radiographic features include root resorption, loss of the root outline, invasion of the root canal, bony expansion, displacement and involvement of adjacent teeth, cortical erosion, and obliteration of the periodontal-ligament space. Surgical excision and related tooth extraction are warranted.

### Cemento-ossifying fibroma

**Figure 5:**
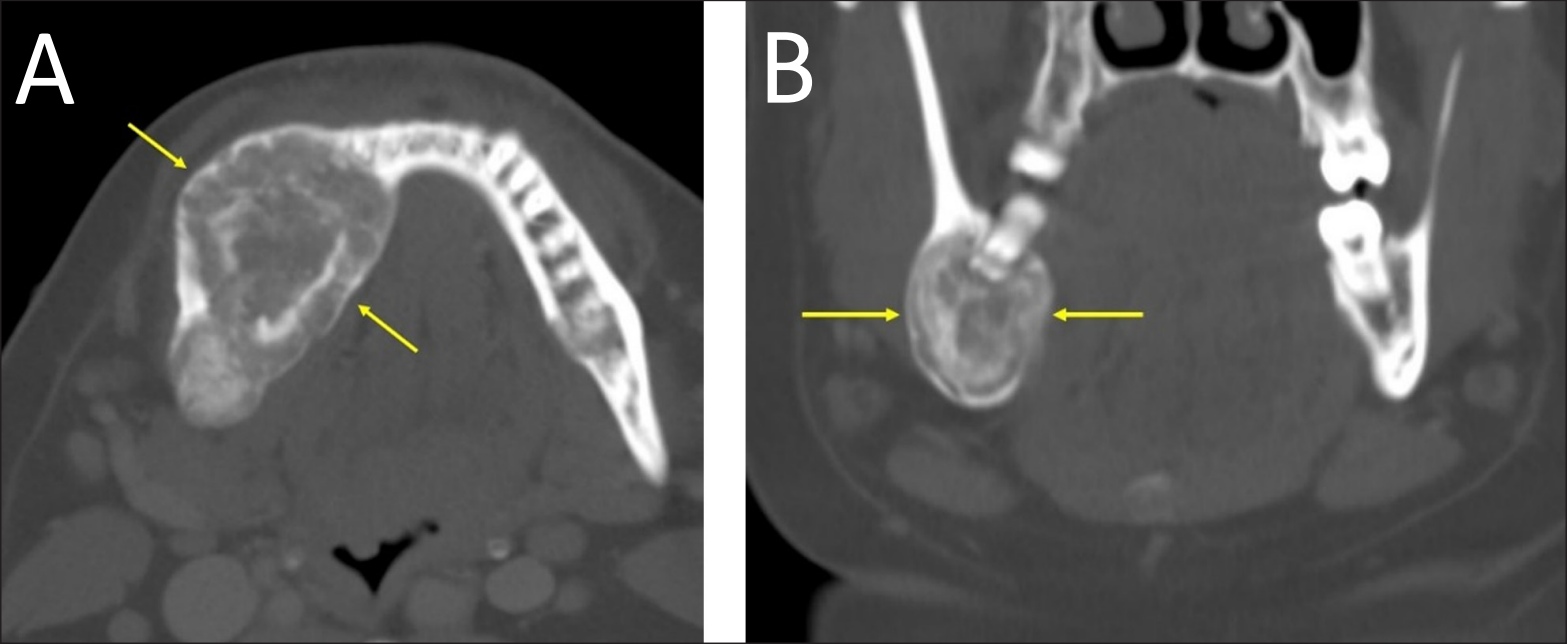
**(A, B)** Cemento-ossifying fibroma. Axial and coronal CT images showing and expansile lesion with sclerotic internal components (arrows).

Cemento-ossifying fibroma is a benign slow-growing fibro-osseous tumor. These tumors are most often seen in women between the third and fourth decades of life and are thought to arise from the periodontal ligament. The growth of the tumor over time may lead to facial asymmetry and the mass causing discomfort or mandibular expansion [11,12]. According to the WHO 2017 Classification of Odontogenic Tumors, they belong to the category of benign odontogenetic tumors, mesenchymal subtype [8,9]. Cemento-ossifying fibromas are composed of varying amounts of cementum, bone, and fibrous tissue. Early stages are radiolucent while lesions containing large amounts of cementum or bone exhibit markedly increased density, typically with the presence of sclerotic components within an expansile lytic lesion (***Figure 5***). The mandibular canal may be displaced inferiorly, whereas in fibrous dysplasia the canal may be displaced in any direction [13,14]. Therapy consists of curettage and enucleation.

## Non-odontogenic sclerotic lesions

### Idiopathic osteosclerosis

**Figure 6:**
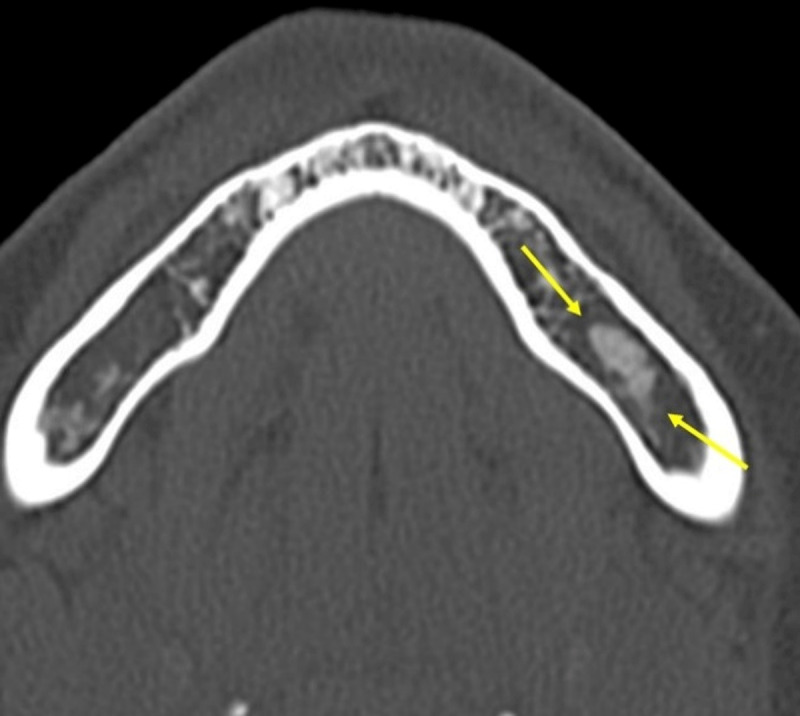
Idiopathic osteosclerosis. Axial CT showing a sclerotic lesion with somewhat spiculated margins (arrows). No low-attenuation rim is seen.

Idiopathic osteosclerosis is an asymptomatic, nonexpansile, localized radiopaque lesion observed in the alveolar process in posterior regions without any obvious etiological agent [15]. Being asymptomatic, it is not associated with inflammation and may remain static or demonstrate slow growth that usually stops when the patient reaches skeletal maturity. Histologically, the lesions consist of dense lamellar bone. Imaging features consist of a well-defined radiopaque, round, elliptical or spiculated mass, usually located in the alveolar process of posterior regions (***Figure 6***). A low-attenuation rim is not seen. No treatment is required.

### Bone island (enostoma)

Dense bone islands, or enostomas, are common incidental findings, consisting of failure of resorption of secondary spongiosa within the trabecular bone [14,16]. Unlike idiopathic osteosclerosis, a bone island has no specific relationship with the dentition. A typical dense bone island has spiculated margins. It is interesting to note that CT attenuation measurements can be useful to distinguish untreated osteoblastic metastases from enostomas: A mean attenuation of 885 HU and a maximum attenuation of 1,060 HU provide reliable thresholds below which a metastatic lesion is the favored diagnosis [16].

### Torus mandibularis, torus palatinus, and torus maxillaris

Tori are protuberances of dense cortical bone that most commonly arise in adults. Occasionally, they contain a small amount of marrow. They are characterized by slow growth that usually arrests spontaneously.

**Figure 7:**
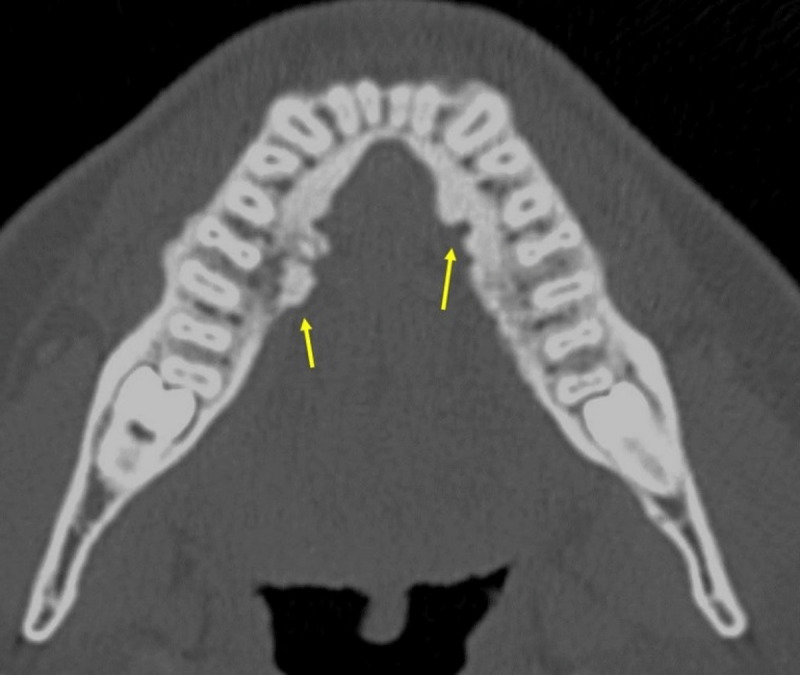
Axial CT showing torus mandibularis.

Torus mandibularis has typical imaging features and arises above the mylohyoid line, along the lingual surface of the mandible (***Figure 7***) [5].

A torus palatinus is a similar nodular bony overgrowth at the midline of the hard palate [5].

A torus maxillaris arises at the lingual surface of the posterior maxilla [1].

### Fibrous dysplasia

**Figure 8:**
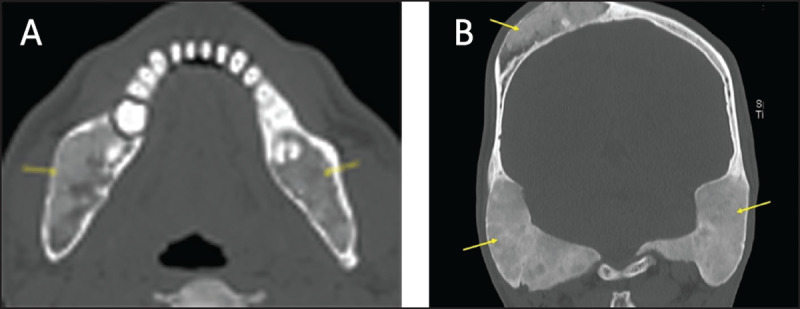
**(A, B)** Polyostotic fibrous dysplasia. Axial (a) and coronal (b) CT showing fibrous dysplasia lesions with typical ground glass appearance in the mandibles and skull bones (arrows).

Fibrous dysplasia is a benign developmental disorder characterized by a dysplastic process of altered osteogenesis with subsequent substitution of normal bone by fibrous tissue that undergoes abnormal mineralization. It typically occurs in the first to third decades of life and is clinically characterized as an asymptomatic bone swelling. The maxilla is involved more frequently than the mandible. Fibrous dysplasia is typically unilateral and may occur within a single bone (monostotic form) or, more often in multiple bones (polyostotic form). Typically, the lesion is radiopaque with ground glass appearance (***Figure 8***) [17,18]. Its cortex remains intact and is often thickened and sclerotic.

### Osteoma

Osteoma is a benign neoplastic lesion, characterized by persistent slow growth. It is composed of mature bone structures with characteristics of cancellous or compact bone.

Osteomas most commonly arise in the craniofacial bones. The most common location in the jaw is the posterior mandibular body or condyle. Multiple osteomas may be associated with Gardner syndrome. Radiologically, osteomas present as a well-defined round radiopaque mass with no radiolucent halo [19]. Osteomas can be either solitary or multiple as found in the Gardner Syndrome, where they are associated with soft tissue tumors, gastro-intestinal polyps and multiple surnumerary impacted teeth.

### Osteoid osteoma

Osteoid osteoma is very rare in the jaw. It is often painful, most pronounced at night, and mitigated by treatment with salicylates. It presents as a lytic lesion with a radiopaque central nidus surrounded by a sclerotic bony margin [20].

### Osteochondroma

Osteochondromas are rarely seen at the craniofacial bones. These tumors have a predilection for the coronoid and condylar processes of the mandible that may exhibit a focal mushroom-shaped enlargement. Larger lesions may cause a decreased mouth opening. Continuity of the cortex and the medullary bone of the lesion and the underlying host bone is a characteristic imaging finding [14].

### Metastases

**Figure 9:**
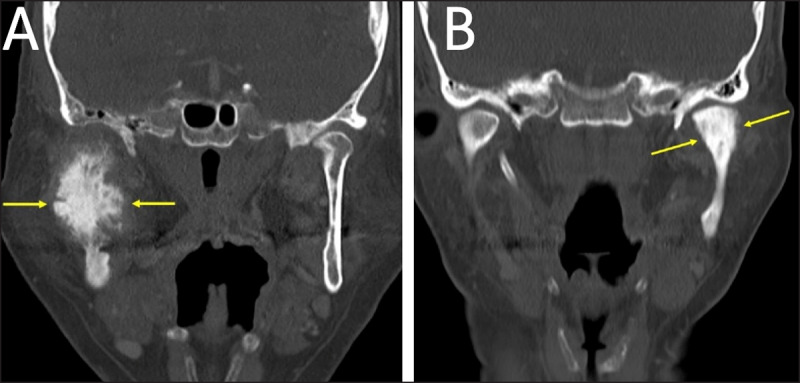
**(A, B)** Sclerotic mandibular metastases. Coronal CT obtained in two different patients showing metastases of prostate cancer (a) and breast cancer (b) (arrows).

The jaws and mouth are uncommon sites for metastatic dissemination. Nevertheless, the incidence of metastatic tumors to the jaws is probably higher than suggested; micrometastatic foci in the jaws were found in 16% of autopsied carcinoma cases despite the absence of radiologic findings [21]. All types of tumors may metastasize to the mandible. Tumors that prefer the jawbone as their metastatic target include prostatic cancer, breast cancer, adrenal cancer, and thyroid cancer. Sclerotic mandibular metastases may be either ill- or well-defined as is the case elsewhere in the body (***Figure 9***).

### Osteosarcoma

Osteosarcoma of the jawbones account for up to 10% of all osteosarcomas. They tend to occur in an older age group usually between 30 and 39 years and have an overall better prognosis than conventional osteosarcoma. Early-stage osteosarcoma may be osteolytic. More advanced lesions are typically osteoblastic or more commonly mixed on radiographs and CBCT due to the presence of mineralized osteoid matrix. Typical features are poorly defined margins, cortex destruction, and aggressive periosteal reaction [14].

## Mixed lytic – sclerotic lesions

### Osteoradionecrosis

Mandibular osteoradionecrosis (ORN) is a serious complication of radiation therapy for neoplasms of the head and neck area. It has a widely varying reported incidence of 5% to 22%. Bone necrosis may occur secondary to hypoxia, hypovascularity, hypocellularity, and fibrosis.

**Figure 10:**
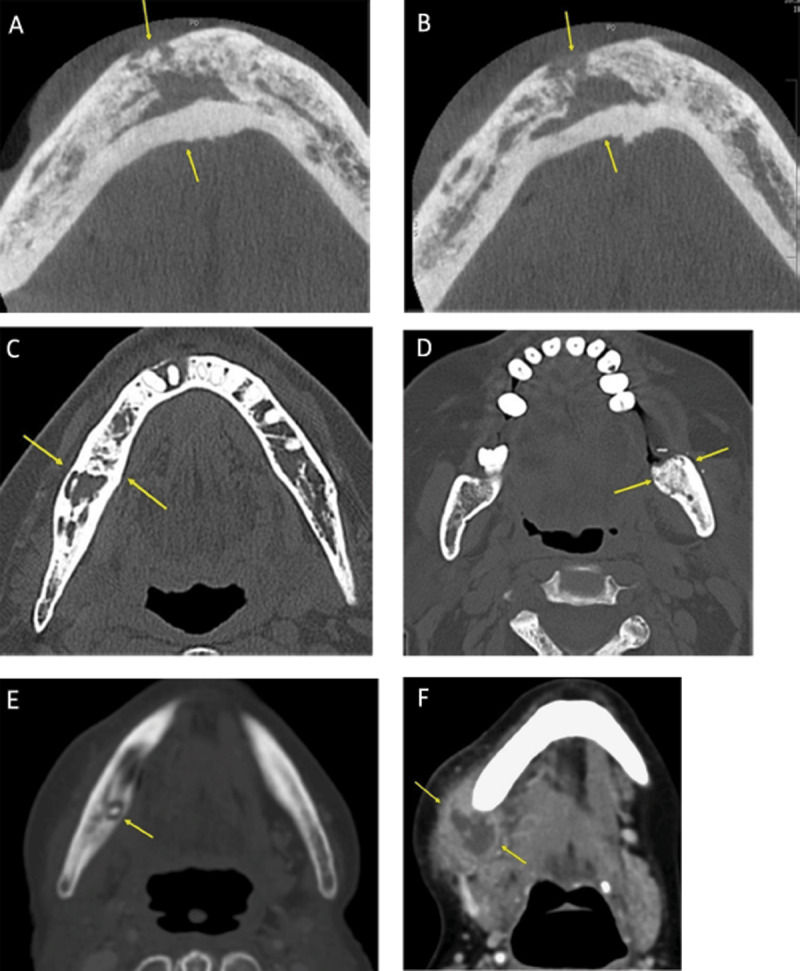
**(A, B)** Mandibular osteoradionecrosis. Axial CT images showing extensive areas of mandibular sclerosis with a loss of trabeculation in spongiosa, and cortical interruptions (arrows). **(C, D)** Bisphosphonate-related osteonecrosis of the jaw (BRONJ) in 2 different patients. Axial CT images shows predominant right-sided sclerosis with interspersed osteolytic areas (c), and predominant left-sided sclerosis with pathologic fracture (d) (arrows). **(E, F)** Mandibular osteomyelitis in an elderly woman who underwent dental extraction. (e) Axial CT in bone window shows heterogeneous bone density of the right mandible with focal osteolysis and a small sclerotic sequestrum. (f) Contrast-enhanced axial CT image in soft tissue window shows an associated submandibular abscess (arrows).

The diagnosis of mandibular ORN is primarily based on clinical symptoms in the appropriate therapeutic setting. The mandible is involved more frequently than the maxilla, probably because of its less robust blood supply. The buccal cortex is more vulnerable than the lingual cortex, although the mandibular body is most commonly affected. At imaging, osteoradionecrosis appears as an area of marked sclerosis with a loss of trabeculation in spongiosa, and cortical interruptions (***Figure 10a, b***) [22]. Other optional imaging features include bone fragmentation or sequestration and areas of gas attenuation in bone with poorly marginated adjacent fluid collections or areas of soft-tissue attenuation. The osseous changes may be associated with significant soft-tissue thickening.

### Biphosphonate-related osteonecrosis

Bisphosphonate-related osteonecrosis of the jaw (BRONJ) is associated with the use of oral or intravenous bisphosphonates to treat various bone conditions such as osteoporosis, multiple myeloma, metastasis, and Paget disease. Bisphosphonates inhibit endothelial proliferation and interrupt intraosseous circulation. Osteonecrosis may be spontaneous or may be precipitated by a traumatic procedure such as tooth extraction or dental surgery. BRONJ should be considered in patients undergoing bisphosphonate therapy with findings of bone necrosis and no history of radiation therapy. BRONJ is typically painful, but some patients may be asymptomatic. Imaging features are comparable to those seen in mandibular osteoradionecrosis (***Figure 1c, d***) [23]. According to Obinata et al. [24], BRONJ typically presents with more pronounced osteosclerosis. On the other hand, osteolysis and spreading of soft tissue inflammation around the jaws may be more pronounced in osteoradionecrosis.

### Osteomyelitis

Osteomyelitis is much more common in the mandible than the maxilla, which is involved in only 1%–6% of cases because of its rich blood supply. Most patients with mandibular osteomyelitis have a history of antecedent dental caries or dental extractions. Other causes of osteomyelitis include dental or mandibular fractures, osteoradionecrosis and rarely, hematogenous spread of infection. Chronic osteomyelitis, which is characterized by a duration longer than one month, may be complicated by sinuses, fistulae, osseous sequestra, or pathologic fractures. Imaging findings of mandibular osteomyelitis include cortical interruption, sclerotic sequestra in low-attenuation zones, periosteal new bone formation, and areas of gas attenuation (***Figure 10e, f***) [1]. In chronic sclerosing osteomyelitis, periosteal new bone formation may be striking.

## Conclusion

Imaging has an important role in detection of lesions of the jaws. For characterization purposes, a systematic analysis is mandatory.

When a sclerotic lesion is observed in the jaw, attention should first be focused on whether and how the lesion is related to a tooth. If a lesion is intimately associated with a tooth, the lesion is most likely odontogenic and the number of possible diagnoses is limited. Moreover, the specific location of the lesion (e.g., periapical or pericoronal) may suggest a specific diagnosis. Cementoblastoma for instance, invariably has a periapical location, while complex odontoma has similar morphological features, and it is typically seen in a pericoronal position.

Besides density and relationship with dentition, other patient and imaging related features may assist in formulating a differential diagnosis or specific diagnosis. These features include the patient’s age, sex, and race, location of the lesion in the jaw (mandible versus maxilla, anterior versus posterior quadrant), and imaging features such as demarcation, cortical involvement, periosteal reaction, and soft tissue changes.

In some cases, lesion characterization may be obtained by imaging alone, particularly in benign lesions.

Even in cases where such characterization is not possible based on imaging features, the radiologist plays an important role and has to determine whether the lesion can be regarded as a “do not touch lesion” or be referred for biopsy.
